# Surgical Planning for the Treatment of a Patient with Multiple, Secondary, Intracranial Echinococcal Cysts

**DOI:** 10.1055/s-0035-1570317

**Published:** 2015-12-18

**Authors:** Emmanouel Chatzidakis, Panagiotis Zogopoulos, Theofilos S. Paleologos, Nikolaos Papageorgiou

**Affiliations:** 1 Department of Neurosurgery, General Hospital of Nikaia-Piraeus “Agios Panteleimon,” Athens, Greece

**Keywords:** hydatid cyst, cerebral cyst, echinococcosis, surgical treatment, surgical planning

## Abstract

A 27-year-old man with a 2-year history of recurrent hospitalizations for various neurologic and cardiologic emergencies was admitted to our hospital presenting with left hemiparesis, which gradually progressed to quadriparesis, bilateral hemianopsia, intracranial hypertension syndrome, and seizures. A diagnosis of echinococcosis was made, based on the radiologic findings of multiple cerebral hydatid cysts and a sizable cyst of the heart. The hydatid cyst of the heart was treated first with a thoracotomy, and after a month he underwent three consecutive surgical operations for the removal of six cerebral cysts. The patient was on albendazole treatment throughout the entire hospitalization, and this led to the shrinkage and finally to the disappearance of two other cerebral cysts. The patient's focal neurologic signs eventually disappeared, with the exception of a slight unilateral hemianopsia. Ten years after his discharge, he remains in a good condition, with no signs of clinical or radiologic relapse.

Hydatid disease (echinococcosis) is a common parasitic infection, mainly in sheep-raising areas of the world (Central Europe, Russia, Turkey, Japan, China, Africa, Australia, the Mediterranean countries, the Middle East, and South America). 1 There are three types of echinococcosis in humans: cystic echinococcosis caused by *Echinococcus granulosus* , alveolar echinococcosis caused by *Echinococcus multilocularis* , and polycystic echinococcosis caused by *Echinococcus vogeli* and/or *Echinococcus oligarthrus* . The liver, lungs, and brain are predominantly involved. 1 The liver is the primary focus of the disease. Cerebral involvement is rare, with its primary form comprising only 0.5 to 3% of all reported hydatid cysts. 2 3 Without timely diagnosis and therapy, the prognosis is dismal, with death the eventual outcome in most cases. 4

## Clinical Manifestation

The diagnosis of cerebral parasitoses depends on the causative agent. Adults or larvae of helminths or protozoa enter the central nervous system and cause meningitis, encephalitis, ventriculitis, myelitis, ischemic stroke, bleeding, venous thrombosis, or cerebral abscess, clinically manifesting as headache, epilepsy, weakness, cognitive decline, impaired consciousness, confusion, coma, or focal neurologic deficits. Headache and vomiting have been reported as the most common initial symptoms. 5 Available diagnostic tools include examination at clinical presentation, blood tests (eosinophilia, plasmodia in blood smear, antibodies against the parasite), cerebrospinal fluid investigations, imaging findings, and occasionally cerebral biopsy. 6

## Radiologic Findings

The diagnosis is usually based on findings at radiologic imaging and in serologic analyses. Because echinococcal lesions can occur almost anywhere in the body, familiarity with the spectrum of cross-sectional imaging appearances is advantageous. Echinococcal lesions may produce widely varied imaging appearances depending on the parasite's growth stage, the tissues or organs affected, and the presence of associated complications. Although the liver is the initial site of mass infestation by *E. multilocularis* , the parasite may disseminate from there to other organs and tissues, such as the lung, heart, brain, bones, and ligaments. Cross-sectional imaging is crucial for differentiating echinococcosis from malignant processes; computed tomography (CT) is most useful for depicting the peripheral calcifications surrounding established echinococcal cysts, and magnetic resonance imaging (MRI) is most helpful for identifying echinococcosis of the central nervous system. 4 Round and thin-walled, homogeneous, low-density, cystic lesions without surrounding edema and enhancement are the main findings on CT in patients with intraparenchymal hydatid cysts. On MRI, the hydatid cyst presents as a round, low-signal lesion in T1-weighted images and a high-signal lesion in T2-weighted images, without enhancement after contrast media injection. 5

## Treatment

Treatment relies on drugs and sometimes surgery. The outcome of cerebral parasitoses is highly variable, depending on the effect of drugs, whether they are self-limiting (e.g., *Angiostrongylus costaricensis* ) or whether they remain undetected or asymptomatic, like 25% of neurocysticercosis cases. 6 The mechanism of recurrence remains unclear (primary infestation, dissemination after spontaneous or intraoperative cyst rupture, or new infestation). 7 Surgical treatment should be considered whenever possible. 8

## Case Report

**Fig. 1:**
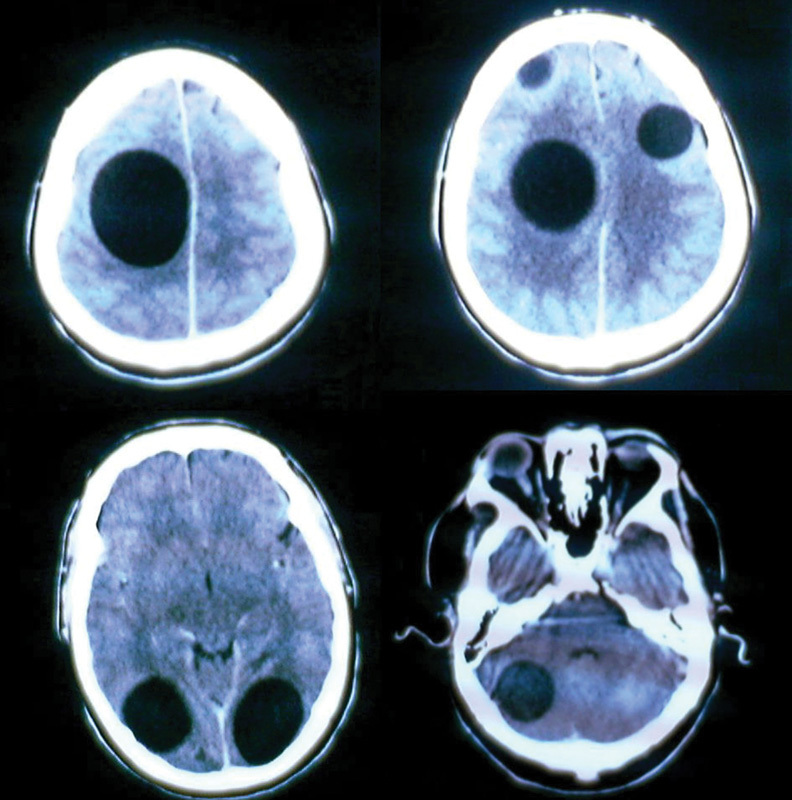
Preoperative cerebral computerized tomography scans revealing multiple hydatid cysts.

A 27-year-old man with a 2-year history of recurrent hospitalizations for various neurologic and cardiologic emergencies was admitted to our hospital presenting with left hemiparesis, which gradually progressed to quadriparesis, bilateral hemianopsia, intracranial hypertension syndrome, and seizures. A diagnosis of echinococcosis was made, based on the radiologic findings of multiple cerebral hydatid cysts and a sizable cyst of the heart ( Fig. 1 ). The hydatid cyst of the heart was treated first with a thoracotomy, and after a month, he underwent three consecutive surgical operations for the removal of the six cerebral cysts. The patient was on albendazole treatment throughout his hospitalization, and this led to the shrinkage and finally to the disappearance of two other cerebral cysts.

**Fig. 2:**
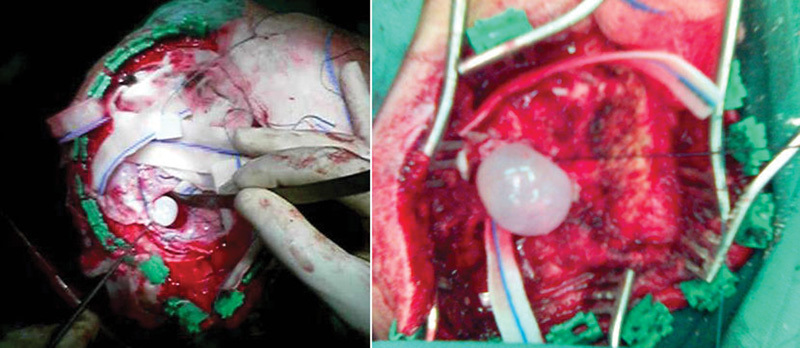
Intraoperative pictures showing removal of hydatid cysts.

**Fig. 3:**
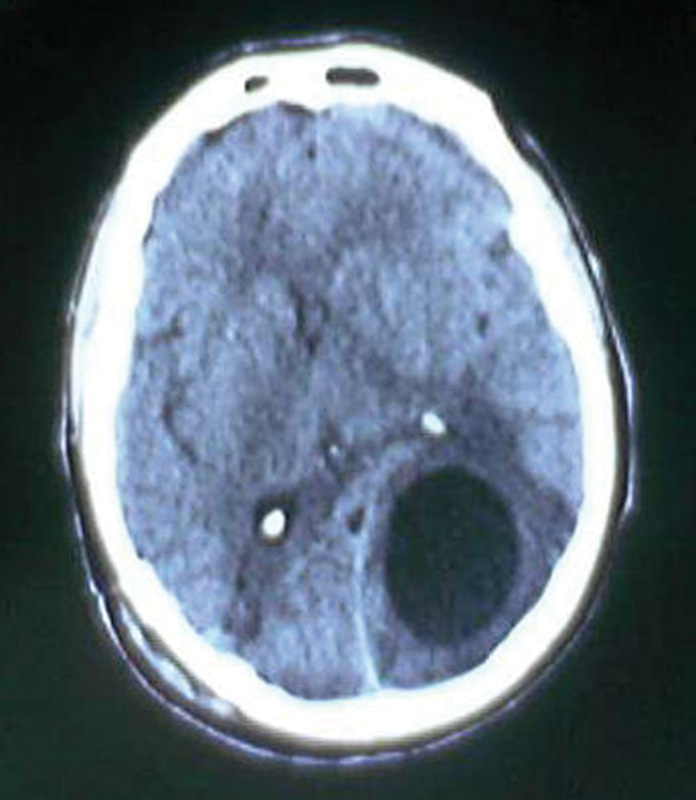
Emergent cerebral computed tomography scan revealing marked expansion of the left occipital lobe cyst with perifocal edema.

**Fig. 4:**
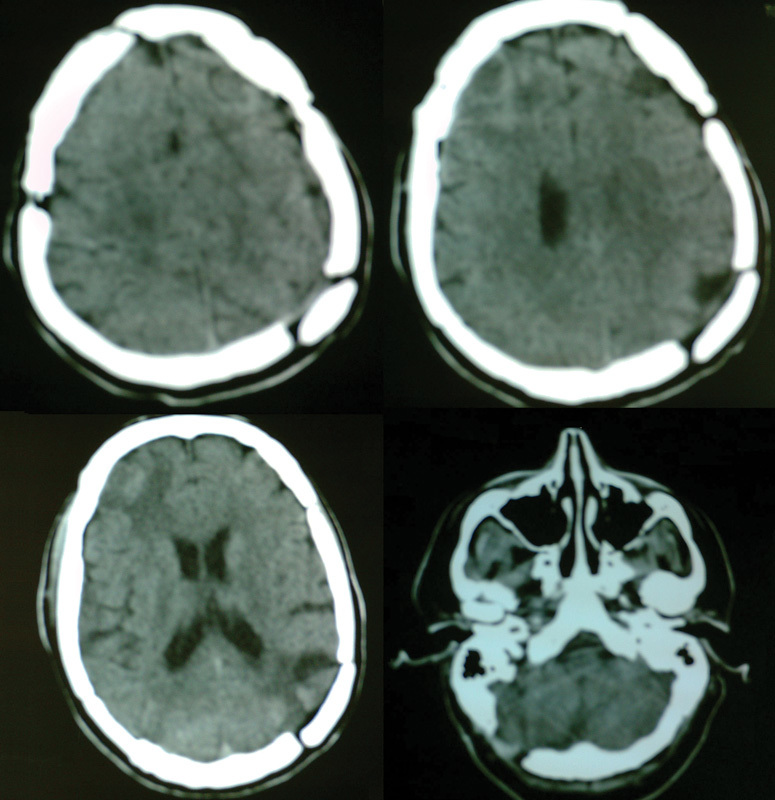
Postoperative cerebral computed tomography scans.

During the first surgical procedure, two frontal hydatid cysts were completely removed via a right frontal craniotomy and another cyst of the occipital lobe was removed via a separate right occipital craniotomy ( Fig. 2 ). The patient initially had an uneventful postoperative recovery but 2 weeks later, while the second operation was being planned, he presented with generalized seizures (with loss of consciousness and urinary incontinence). Emergent cerebral CT scan revealed a marked expansion of the left occipital lobe cyst, cerebral edema, and midline shift ( Fig. 3 ). A possible explanation of this event is that the removal of the contralateral (right occipital) cyst disturbed the pressure balance between these two lesions and allowed the expansion of the left occipital cyst toward the right side. The patient was emergently operated on and a left occipital craniotomy was performed for the complete removal of the left occipital cyst. There were no postoperative events, and 10 days later the third procedure was performed. A right cerebellar hemisphere cyst was removed via a right suboccipital craniectomy, and a left frontal lobe cyst was removed via a left frontal craniotomy ( Fig. 4 ).

The patient's focal neurologic signs eventually disappeared, with the exception of a slight unilateral hemianopsia. Ten years after his discharge, he remains in a good condition, with no signs of clinical or radiologic relapse.

## Discussion

The decision regarding the initial surgical approach (and hence, which hydatid cysts would be the first to be removed) was based on clinical presentation, characteristics (localization and size of the cysts) affecting the morbidity of the disease, and the risk of potential major intracranial events (mass effect and imminent cerebral herniation). We therefore decided to perform two separate procedures, the first in the right hemisphere where the largest cyst was located and the second in the left hemisphere, because the multiplicity of the cysts in both hemispheres would render their surgical removal in one setting extremely lengthy and toilsome.

However, a sudden clinical deterioration of the patient 2 weeks after the first operation made an emergent surgical procedure necessary before the planned contralateral side approach. Τherefore, neurosurgeons attempting surgical removal of such multiple space-occupying lesions should always bear in mind that by doing so they may disturb an established pressure balance inside the cranial vault and thus contribute to an imminent herniation.

It is noteworthy that the cyst fluid in all cases was crystal clear, and microbiological tests of both cyst fluid and cyst wall were negative for parasites. This points to the importance of antiparasitic treatment. Albendazole in this case proved extremely efficient because it led to the shrinkage and finally to the disappearance of two other small cerebral hydatid cysts.

## Conclusions

Cerebral hydatid cyst disease should be kept in mind in the differential diagnosis of increased intracranial pressure syndrome, especially among patients in areas endemic for echinococcosis. 2 CT and MRI are the best diagnostic methods, and surgery is the treatment of choice for intracranial hydatid cysts. 5 Cases of multiple intracranial hydatid cysts are extremely rare in the literature, and the surgical planning and approach presented here might prove beneficial to physicians in the future.
